# Perspective for Future Medicine: Multidisciplinary Computational Anatomy-Based Medicine with Artificial Intelligence

**DOI:** 10.34133/2021/9160478

**Published:** 2021-01-08

**Authors:** Makoto Hashizume

**Affiliations:** Kyushu University and Kitakyushu Koga Hospital, Japan

## Abstract

Multidisciplinary computational anatomy (MCA) is a new frontier of science that provides a mathematical analysis basis for the comprehensive and useful understanding of “dynamic living human anatomy.” It defines a new mathematical modeling method for the early detection and highly intelligent diagnosis and treatment of incurable or intractable diseases. The MCA is a method of scientific research on innovative areas based on the medical images that are integrated with the information related to: (1) the spatial axis, extending from a cell size to an organ size; (2) the time series axis, extending from an embryo to post mortem body; (3) the functional axis on physiology or metabolism which is reflected in a variety of medical image modalities; and (4) the pathological axis, extending from a healthy physical condition to a diseased condition. It aims to integrate multiple prediction models such as multiscale prediction model, temporal prediction model, anatomy function prediction model, and anatomy-pathology prediction model. Artificial intelligence has been introduced to accelerate the calculation of statistic mathematical analysis. The future perspective is expected to promote the development of human resources as well as a new MCA-based scientific interdisciplinary field composed of mathematical statistics, information sciences, computing data science, robotics, and biomedical engineering and clinical applications. The MCA-based medicine might be one of the solutions to overcome the difficulties in the current medicine.

## 1. Main Text

The absolute necessary condition for making a decision on clinical diagnosis and treatment is to better understand each patient's physiological and pathological dynamics, including morphological and functional information. It is true even in such basic sciences as cyborg and bionic system.

The medical images with a variety of modalities are helpful for us to better image and understand the changes of the whole living human body over time in a 3D space. A better understanding of anatomical dynamics might be more helpful to solve the difficulties in current medicine. The modeling of Personalized Digital Patient is the most important factor in surgical data science [1], allowing us to understand the patient's anatomy or pathogenesis and to make surgical planning or surgical navigation. “Personalized Digital Patient” or “Digital Twin” is a digital representation of the patient's anatomy and physiology based on the models whose parameters can be learned automatically from real or simulated medical images and additional clinical data, biological behavior data, and environmental data. Then, advanced algorithms can use the learned parameters to support the three pillars of digital medicine: diagnosis, prognosis, and therapy [2]. The innovative discoveries in diagnosis and treatment might be produced by the ideas coming from the new approaches to previous unmeasurable information.

The origin of computational anatomy can be traced back to about a century ago, when the famous book on Shape and Growth by D' Arey Wentworth Thompson was published in 1917 [3]. It showed the importance of physical laws and mechanics as the fundamental determinants of the form and structure of the living organisms, while demonstrating that the differences in the forms of related animals could be explained by relatively simple mathematical transformations. Modern computational anatomy is emerging as a discipline focused on the quantitative analysis of the variability in organ shape and on the application of this analysis to computer-aided diagnosis (CAD) and computer-aided surgery (CAS). Reflecting on these developments, we can see that advanced computational anatomy has provided a technical platform for the better understanding of anatomic variability, an aid to the diagnosis of disease, and a means to simulate surgical interventions. Advanced statistical method is required based on multimodality images as well as other clinical data of a dynamic living human body.

**Figure 1:**
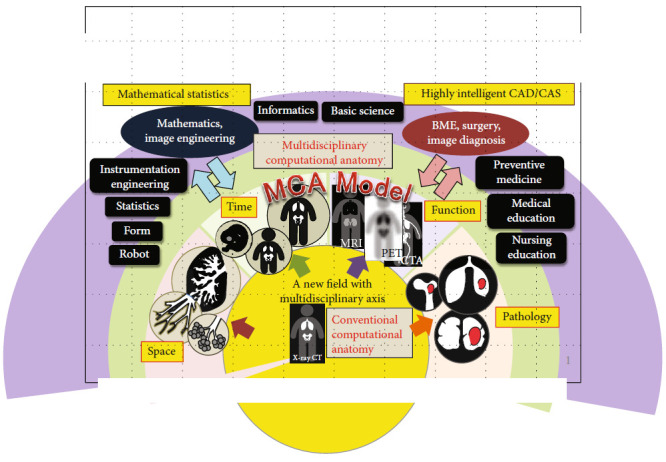
A new field of multidisciplinary computational anatomy. A variety of medical images are integrated with the information along the spatial, temporal, functional, and pathological axes. It allows us to better understand the medical image of each patient, who, as a whole, is a dynamic living human.

A new model of multidisciplinary computational anatomy (MCA) [4] must be established for the innovative advanced technology based on mathematical, engineering, and medical sciences. It aims to find the significant meaning and statistical relationships among the different modalities of medical images, to integrate a large amount of information on human structure and life phenomenon, to make different kinds of information visible, and to establish the principles and models for mathematical statistic methodology in order to access or search for the information more easily. The integrated multidisciplinary information is applied to medical judgment or decision-making by completely understanding the anatomy and to the practical planning before action. It is a new frontier of science that provides a mathematical analysis basis for the comprehensive and useful understanding of “dynamic, living human anatomy” (see Figure 1). It defines a new mathematical method for the early detection and highly intelligent diagnosis and treatment of incurable or intractable diseases. The MCA is a method of scientific research on innovative areas based on the medical images that are integrated with the information related to: (1) the spatial axis, extending from a cell size to an organ size; (2) the time series axis, extending from an embryo to post mortem body; (3) the functional axis on physiology or metabolism which is reflected in a variety of medical image modalities; and (4) the pathological axis, extending from a healthy physical condition to a diseased condition. The task is to establish multiple prediction models such as multiscale prediction model, temporal prediction model, anatomy-function prediction model, and anatomy-pathology prediction model. In MCA, artificial intelligence (AI) has been introduced to accelerate the calculation of statistic mathematical analysis.

Sato et al. investigated the MCA modeling for musculoskeletal system, in which the physiological units in functional (muscle fiber) transmission systems, including connective tissues and physical connections, are modeled [5]. The new discipline provides a modeling framework for personalized functional musculoskeletal anatomy, which was not available before. Mori et al. focused on the development of a method that supports the seamless understanding of multimodal and multiscale medical images [6]. They developed the following technologies: (a) multidimensional seamless registration, (b) the analysis of multidimensional seamless anatomical structure, (c) the understanding and annotation of multidimensional meta-anatomical structure, and (d) the assistance in making a decision on diagnosis and surgery. The deep-learning technique was widely used to improve the image analysis performance. An automated real-time pathological prediction method was developed for endocytoscopic images [7]. Endocytoscopy has recently been developed as a new endoscopic imaging modality. It provides an ultralarge 520x magnification which allows us to observe the cells and their nuclei on the surface of intestinal mucosal layer. But this new device requires expertise, so it is especially suitable for clinical use. It is expected to contribute to the early detection of colonic neoplastic lesion and to the reduction of cancer incidence and death rate [8]. Suzuki et al. developed a 4-dimensional human body model capable of deforming the skin and internal structures (organs, skeletal muscles, vasculature, etc.) with respect to human body movement [9]. The body structure in the human model was a T1 weighted image with a slice thickness of 2 mm. Suzuki et al. segmented the subject's MRI image and the shapes of body surface, major organs, skeletal system, and vascular system and built the surface models based on these data.

A larger population has undertaken oral and maxillofacial surgery (OMS) for therapeutic and aesthetical purposes. The surgeon is under heavy physical and mental burden when conducting the precise OMS procedures such as drilling a screw hole on the jaw bone. Therefore, some surgical navigation methods and a surgery-assisting robot have been proposed to guide the operation and relieve the surgeon's workload. Kobayashi et al. developed a marker-based/marker-less AR navigation system [10] and a compact OMS robot for precise positioning [11]. They seamlessly integrated these two systems and developed an autonomous surgical system [12]. As a result, the roles of surgeon and surgical system could be exchanged, that is, the robot was the primary operator and the surgeon was the surveillant. Kobayashi et al. proposed the use of an intelligent autonomous surgical robot to approach the affected surgical area in accordance with the MCA model and limited intraoperative biological information.

The MCA-based medicine will promote the development of human resources as well as a new MCA-based scientific interdisciplinary field composed of mathematical statistics, information sciences, computing data science, robotics, and biomedical engineering and clinical applications. The MCA-based medicine might be one of the best solutions to overcome the difficulties in the current medicine. More effective and safer methodologies will be further developed in an innovative manner to obtain a healthy lifestyle.

## Data Availability

The data used to support the findings of this study are available from the corresponding author upon request.
